# Mathematical modelling of vector-borne diseases and insecticide resistance evolution

**DOI:** 10.1186/s40409-017-0123-x

**Published:** 2017-07-06

**Authors:** Maria Laura Gabriel Kuniyoshi, Fernando Luiz Pio dos Santos

**Affiliations:** Department of Biostatistics, Institute of Biosciences of Botucatu, São Paulo State University (UNESP - Universidade Estadual Paulista), Street Prof. Dr. Irina Delanova Gemtchujnicov, no number, Rubião Júnior, zip code 18618-693, PO box 510, Botucatu, SP Brazil

**Keywords:** Epidemiology, Population genetics, Tropical diseases, Insecticides, Theoretical modelling, Numerical simulation, ODE system

## Abstract

**Background:**

Vector-borne diseases are important public health issues and, consequently, in silico models that simulate them can be useful. The susceptible-infected-recovered (SIR) model simulates the population dynamics of an epidemic and can be easily adapted to vector-borne diseases, whereas the Hardy-Weinberg model simulates allele frequencies and can be used to study insecticide resistance evolution. The aim of the present study is to develop a coupled system that unifies both models, therefore enabling the analysis of the effects of vector population genetics on the population dynamics of an epidemic.

**Methods:**

Our model consists of an ordinary differential equation system. We considered the populations of susceptible, infected and recovered humans, as well as susceptible and infected vectors. Concerning these vectors, we considered a pair of alleles, with complete dominance interaction that determined the rate of mortality induced by insecticides. Thus, we were able to separate the vectors according to the genotype. We performed three numerical simulations of the model. In simulation one, both alleles conferred the same mortality rate values, therefore there was no resistant strain. In simulations two and three, the recessive and dominant alleles, respectively, conferred a lower mortality.

**Results:**

Our numerical results show that the genetic composition of the vector population affects the dynamics of human diseases. We found that the absolute number of vectors and the proportion of infected vectors are smaller when there is no resistant strain, whilst the ratio of infected people is larger in the presence of insecticide-resistant vectors. The dynamics observed for infected humans in all simulations has a very similar shape to real epidemiological data.

**Conclusion:**

The population genetics of vectors can affect epidemiological dynamics, and the presence of insecticide-resistant strains can increase the number of infected people. Based on the present results, the model is a basis for development of other models and for investigating population dynamics.

**Electronic supplementary material:**

The online version of this article (doi:10.1186/s40409-017-0123-x) contains supplementary material, which is available to authorized users.

## Background

Vector-borne diseases represent one sixth of the sicknesses suffered by the global population, and more than 50% of the world is at risk of coming down with them [1]. One of the most common vector-borne diseases is dengue fever, as 2.5 billion people from more than 100 countries are infected with this illness [2]. Dengue is a febrile infectious disease caused by a virus of the family *Flaviridae*, which has four distinct serotypes: DEN1, DEN2, and DEN3 DEN4. The transmission mechanism is through bites of female mosquitos from the genus *Aedes*, especially of the species *Aedes aegypti* [3, 4]. Rapid and unorganized urban growth, which is related to a country’s development and rural flight, contributes to mosquito proliferation and, consequently to the epidemic of this urban disease. A serious characteristic is that *Aedes aegypti* can also transmit the zika and chikungunya virus. The triple epidemic dengue-zika-chikungunya coexists in many endemic areas [5]. Despite all efforts, developing new tools and improving the existing strategies is very important, necessary and still far from ideal.

Therefore, it is relevant to create models to simulate the phenomenology of epidemic diseases and to understand the respective dynamics. This kind of in silico research, performed entirely on computer, is supposed to be very advantageous. As mathematical modelling and numerical simulation involve no laboratorial experiments, they imply economy of time and resources, and can even substitute pilot experiments [6]. Furthermore, as they involve no live models, there are no bioethical restraints, so their association with in vivo and in vitro experiments can reduce the number of animals that would undergo stressful and harmful situations [6].

**Fig. 1:**
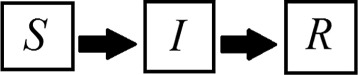
Classical SIR model scheme. The susceptible population becomes infected, which, in turn, becomes recovered. *S* = susceptible; *I* = infected; *R* = recovered

The susceptible-infected-recovered (SIR) model was firstly proposed by Kermack and McKendrick [7], and since then many studies based on their idea have been developed. This model enables scientists to simulate the dynamics of a population under an epidemic of an infectious disease over time. Three classes of individuals are considered (Fig. 1): susceptible, those who are at risk of contracting the disease; infected, those people who have the pathogen in their organism and are able to transmit it; and recovered, those who have been cured and are immune to the illness [7]. The original SIR model focuses only on host-to-host transmission [7]. However, there is a useful class of SIR models to study vector-borne diseases [8]. For instance, Bailey [9] described the susceptible-infected-recovered/susceptible-infected (SIRSI) model, in which the host population is divided into susceptible, infected and recovered, and the vector population into susceptible and infected.

Another important field of research related to vector-borne diseases is population genetics, specifically studies regarding the selection of insecticide-resistant vector strains [10]. Population genetics is the branch of biology that investigates frequencies of alleles and genotypes in populations [11]. Resistant strains of vectors are those that resist and survive the effects of insecticides, and usually have their origin in genetic causes [12]. Positive selection of particular phenotypes, such as insecticide resistance, occur because they confer a long-term survival or reproduction rate (*i.e.*, a greater fitness to the environment), and therefore individuals that express them have more chance of transmitting their genes to the next generation. Subsequently, the frequency of the allele which determines resistance increases over time, and so does the proportion of individuals that portray this gene [13]. This situation is an example of the natural selection, proposed by Darwin [14]. The importance of understanding the genetics of vector insecticide resistance is emphasized in the Global Plan for Insecticide Resistance Management from the World Health Organization, in which the relevance of theoretical modelling is also highlighted [12].

In population genetics, another important concept is the Hardy-Weinberg equilibrium. Its conditions are the absence of evolutionary factors, including the natural selection. To make it possible, all the allelomorphs and genotypes must confer the same fitness, so that the allele frequencies remain constant over time [15]. In addition, the pattern of inheritance must be Mendelian; the proportion of males and females must be equal; and the population must be panmictic and large [16]. Considering only two alleles, the Hardy-Weinberg equilibrium is achieved when the proportions of the genotypes remain constant and are represented by the following equation [1]:

1 $$  p^{2}+2pq+q^{2}=1  $$

Where *p* and *q* are the frequencies of the dominant and the recessive allele, respectively, and *p*+*q*=1. *p* ^2^, 2*p* *q* and *q* ^2^ are the proportions of pure dominants, heterozygotes and pure recessives, and also sum 1.

Considering all this information, it is important to highlight that studying the alleles which confer resistance to the vectors might influence various aspects of control strategies, such as the usage of insecticides [17]. For example, in Zambia, this kind of research determined that pyrethroids should not be used anymore in insecticide rotation strategies for *Anopheles* sp. control [18]. Another example is the work of Luz et al. [19], in which mathematical simulations were used to determine the efficiency of insecticide control against *A. aegypti* and to study the evolution of insecticide resistance. In the model of Luz et al. [19], it is considered that the insecticide resistance is linked to one locus with two alleles, so that *A. aegypti* is divided into three groups according to the genotype. The incorporation of population genetics into mathematical models can be useful for investigation of other aspects as well. For example, Schechtman and Souza [20] developed a deterministic mathematical model based on *A. aegypti* that considers both the concept of Hardy-Weinberg equilibrium and an insecticide resistance gene with Mendelian inheritance pattern. In the research of Schechtman and Souza [20], computational simulations were performed to investigate persistence of vector insecticide resistance and reversion to insecticide susceptibility. Both models might be valuable tools for public health organizations that are responsible for control of vector-borne diseases. However, few studies have investigated mathematical models that couple SIR models and population genetics.

In this study, we propose a mathematical model of ordinary differential equations that represents a vector-borne disease epidemic and connects the main ideas of SIR mathematical models and population genetics. Our model is based on arboviral diseases, specially chikungunya, dengue and yellow fever. Using this approach, we considered the possibility of splitting the vector population according to the genotype into three groups, each of them with different characteristics, represented by distinct values of biological parameters. In our case, we consider different mortality values. Therefore, it is possible to simulate the variation of the genetic composition of vector population over time. There are two primary aims of this study: 1. To investigate whether the dominance of an insecticide resistance gene influences the epidemics dynamics 2. To create a model that integrates epidemiology and population genetics, from which more sophisticated models and new approaches can be developed.

## Methods

We took into account infectious diseases that affect humans and are transmitted by an insect vector, specially arboviral diseases, such as yellow fever and chikungunya.

### General assumptions

First of all, in order to better understand our model, the assumptions we made for its development should be mentioned:

- **Assumption 1**: The breeding in the populations is random, i.e., all the individuals have the same chance of reproduction and they mate with any other individual in the population with the same probability;
- **Assumption 2**: The disease is transmitted only horizontally. Vertical transmission occurs in real situations, but we disregarded it in order to present a clearer model;
- **Assumption 3**: There are no evolutionary factors except for the biological selection (we disregard genetic drift and mutation, for instance), because of the same reason presented in the previous assumption;
- **Assumption 4**: The lifetime span of the vectors is not long enough for the recovery of an infected insect;
- **Assumption 5**: There are no immigrants or emigrants in the populations considered, since considering human mobility would imply additional features, such as separation of population into patches [21], which is not the focus of this investigation.

### Development of the model

#### Human populations

**Fig. 2:**
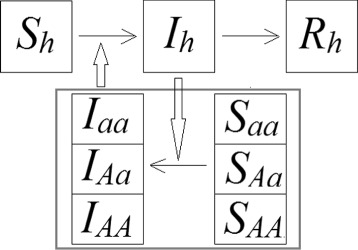
Scheme of the SIRSI model considering genetics aspects of the vector. *AA*, *Aa* and *aa* mean dominant homozygotes, heterozygotes and recessive homozygotes, respectively, for the Susceptible (*S*) and Infected (*I*) mosquito populations. *S*_*h*_, *I*_*h*_ and *R*_*h*_ are the susceptible, infected and susceptible compartments for humans. Vectors become infected when they interact with an infected host; and susceptible humans become infected when they are bitten by an infected vector

In our model, we considered both the populations of humans and vectors. The humans were divided into susceptible, infected and recovered (Fig. 2). New human individuals are introduced into the susceptible population according to expression *c*·*N* *h*, in which *c* is the birth rate and *Nh* is the total human population. Susceptible humans are infected when they are bitten by an infected vector, which happens at a probability *β* *h*. Infected humans recover at a rate *γ*, and the human mortality rate is *μ* *h*, which does not vary with the compartment considered.

#### Population genetics

We take into account a pair of alleles in the vector population. These alleles determine the presence or not of insecticide resistance, and, consequently, the value of the animal’s mortality rate. They hold an interaction of complete dominance. We represented the recessive allelomorph by *a* and the dominant one by *A*. We calculated the frequency of each allele by [22]:

2 $$\begin{array}{*{20}l} f(a)=q=\frac{2N_{aa}+N_{Aa}}{2N_{T}} \end{array} $$

3 $$\begin{array}{*{20}l} f(A)=p=\frac{2N_{AA}+N_{Aa}}{2N_{T}} \end{array} $$

where *N*_*aa*_, *N*_*Aa*_, *N*_*AA*_ and *N*_*T*_ are the populations of recessive homozygotes, heterozygotes, dominant homozygotes, and the total population, respectively.

Considering the frequencies of the alleles, the probability of there being a gamete portraying allele *A* is *p* and allele *a* is *q*. Therefore, the probability of the formation of each genotype during fertilization is given in Table 1 below.

**Table 1:** Probability of formation of genotypes in the next generation

	Probability of a gamete portraying *A*=*p*	Probability of a gamete portraying *a*=*q*
Probability of a gamete portraying *A*=*p*	Probability of the formation of genotype *A* *A*=*p*·*p*	Probability of the formation of genotype *A* *a*=*p*·*q*
Probability of a gamete portraying *a*=*q*	Probability of the formation of genotype *A* *a*=*p*·*q*	Probability of the formation of genotype *a* *a*=*q*·*q*

Consequently, the proportion of the genotypes *aa*, *Aa* and *AA* in the next generation in time *t*+1 can be calculated by *p* ^2^, 2*p* *q* and *q* ^2^, in this order. Considering that *θ* is the vector birth rate and that the total population in the next generation is equal to *N*_*T*_·*θ*, we have the following equations:

4 $$\begin{array}{*{20}l}  N_{aa}^{t+1}=q^{2}\theta {N_{T}^{t}}+N_{aa}^{t} \end{array} $$

5 $$\begin{array}{*{20}l}  N_{Aa}^{t+1}=2pq\theta {N_{T}^{t}}+N_{Aa}^{t} \end{array} $$

6 $$\begin{array}{*{20}l}  N_{AA}^{t+1}=p^{2}\theta {N_{T}^{t}}+N_{AA}^{t} \end{array} $$

Where ${N_{T}^{t}}=N_{aa}^{t}+N_{Aa}^{t}+N_{AA}^{t}$ in time *t* and ${N_{T}^{0}}=N_{aa}^{0}+N_{Aa}^{0}+N_{AA}^{0}$. As we consider that there are no vertical transmission, all new individuals are introduced in the susceptible compartment therefore:

7 $$\begin{array}{*{20}l}  S_{aa}^{t+1}=q^{2}\theta N_{T}^{t} \end{array} $$

8 $$\begin{array}{*{20}l}  S_{Aa}^{t+1}=2pq\theta N_{T}^{t} \end{array} $$

9 $$\begin{array}{*{20}l}  S_{AA}^{t+1}=p^{2}\theta N_{T}^{t} \end{array} $$

#### Vector populations

As we considered a pair of alleles for the vector population, we could separate the vector population into recessive homozygotes, heterozygotes and dominant homozygotes. Each of these compartments were subdivided into susceptible and infected (Fig. 2). New individuals are introduced into the populations through the susceptible compartment, according to what is described in the previous item. Susceptible insects become infected when they bite infected humans, with a probability *β* *v*. This way, *β* *v* represents the proportion of vectors that effectively gets infected. This parameter is particularly useful because field studies demonstrate that only a small percentage of vectors gets infected with the pathogen [23].

The mortality rate has two components: the natural mortality rate (*μ*_*N*_), whose value is equal for all vector groups considered here; and the insecticide-induced mortality rate (*μ*_*I*_), whose value was smaller for the resistant strain and greater for the wild type. Eq. 10 shows the components of the total mortality rate. We considered *μ*_*aa*_, *μ*_*Aa*_ and *μ*_*AA*_ as the total mortality rates for recessive homozygotes, heterozygotes and dominant homozygotes, respectively. As allele *A* is dominant over *a* and we consider a complete dominance interaction, *μ*_*Aa*_ and *μ*_*AA*_ will always have the same value.

10 $$ \mu_{T}=\mu_{N}+\mu_{I}   $$

This way, according to our system, the increase in susceptible vector populations is dependent of allele frequencies and total vector population, and limited by a carrying capacity (*K*). The decrease of susceptible vector population is proportional to its own size and the percentage of vectors that become infected. On the other hand, the increase in infected vector populations is dependent on the proportion of infected humans $\left (\frac {I_{h}}{N_{h}}\right)$ and on the number of susceptible vectors with the correspondent genotype (*S*_*v*_). In contrast, the decrease of infected vector populations is proportional only to its own size, independently of other populations.

#### The system of ordinary differential equations

Our model was based on the one proposed by Santos [21]. The variables and parameters are summarized in Tables 2, 3 and 4. Our mathematical model can be represented by the following system of ordinary differential equations:

11 $$  \left\{ \begin{array}{l} \frac{S_{h}}{dt}=cN_{h}-\left(\frac{\beta_{h}I_{v}}{N_{h}}+\mu_{h}\right)S_{h} \\ \frac{I_{h}}{dt}=\frac{\beta_{h}I_{v}}{N_{h}}S_{h}-\left(\gamma+\mu_{h}\right)I_{h} \\ \frac{R_{h}}{dt}=\gamma I_{h}-\mu_{h}R_{h} \\ \frac{S_{aa}}{dt}=q^{2}\theta N_{v}\left(1-\frac{N_{v}}{K}\right)-\left(\frac{\beta_{v}I_{h}}{N_{h}}+\mu_{aa}\right)S_{aa} \\ \frac{I_{aa}}{dt}=\frac{\beta_{v}I_{h}}{N_{h}}S_{aa}-\mu_{aa}I_{aa} \\ \frac{S_{Aa}}{dt}=2pq\theta N_{v}\left(1-\frac{N_{v}}{K}\right)-\left(\frac{\beta_{v}I_{h}}{N_{h}}+\mu_{Aa}\right)S_{Aa} \\ \frac{I_{Aa}}{dt}=\frac{\beta_{v}I_{h}}{N_{h}}S_{Aa}-\mu_{Aa}I_{Aa} \\ \frac{S_{AA}}{dt}=p^{2}\theta N_{v}\left(1-\frac{N_{v}}{K}\right)-\left(\frac{\beta_{v}I_{h}}{N_{h}}+\mu_{AA}\right)S_{AA} \\ \frac{I_{AA}}{dt}=\frac{\beta_{v}I_{h}}{N_{h}}S_{AA}-\mu_{AA}I_{AA} \\ \end{array}\right.  $$

**Table 2:** Biological parameter values and meanings

Parameter	Meaning	Value	Parameter	Meaning	Value
*C*	Human birth rate	0.457·10^−4^	*μ*_*aa*_	Recessive homozygote vector mortality rate	Table 4
*μ*_*h*_	Human mortality rate	0.457·10^−4^	*μ*_*Aa*_	Heterozygote vector mortality rate	Table 4
*β*_*h*_	Probability of vector-human transmission	0.4	*μ*_*AA*_	Dominant homozygote vector mortality rate	Table 4
*γ*	Human recovery rate	0.121	*β*_*v*_	Probability of human-vector transmission	0.4
*θ*	Vector oviposition rate	6.353	*K*	Carrying capacity	2·10^5^

Values obtained in Santos [21]

**Table 3:** Variable values and meanings

Variable	Meaning	Initial value	Variable	Meaning	Initial value
*S*_*h*_	Human susceptible population	9·10^3^	*S*_*aa*_	Recessive homozygote vector susceptible population	4.75·10^3^
*I*_*h*_	Human infected population	2·10^2^	*I*_*aa*_	Recessive homozygote vector infected population	4.75·10^3^
*R*_*h*_	Human recovered population	3·10^2^	*N*_*aa*_	Recessive homozygote vector total population	9.5·10^3^
*N*_*h*_	Human total population	9.5·10^3^	*S*_*AA*_	Dominant homozygote vector susceptible population	2.375·10^3^
*S*_*Aa*_	Heterozygote vector susceptible population	2.375·10^3^	*I*_*AA*_	Dominant homozygote vector infected population	2.375·10^3^
*I*_*Aa*_	Heterozygote vector infected population	2.375·10^3^	*N*_*AA*_	Dominant homozygote vector total population	4.75·10^3^
*N*_*Aa*_	Heterozygote vector total population	4.75·10^3^	*N*_*v*_	Vector total population	1.9·10^4^
*I*_*v*_	Infected vector population	3·10^4^	*f*_*aa*_	Frequency of recessive homozygotes	$\frac {N_{aa}}{N_{v}}$
*f*_*Aa*_	Frequency of heterozygotes	$\frac {N_{aa}}{N_{v}}$	*f*_*AA*_	Frequency of dominant homozygotes	$\frac {N_{aa}}{N_{v}}$
*q*	Frequency of recessive allele	Eq. 2	*p*	Frequency of dominant allele	Eq. 3

**Table 4:** Values of mortality rates for the three simulations

Allele	Genotypes in which the allele expresses	Mortality rate (day^−1^)		
		Simulation 1	Simulation 2	Simulation 3
Recessive (*a*)	*aa*	0.25	0.01	0.25
Dominant (*A*)	*A* *a*,*A* *A*	0.25	0.25	0.01

### Computational simulations

The simulations were made using the software MatLab^®;^. We ran the discrete form of system 11, which was obtained through the Euler method (Additional file 1). The time, which was measured in days, varied from 0 to 100, and *Δ* *t* was equal to 0.1. The parameter values were obtained from Santos [21] and are shown in Tables 2 and 4. The initial values of the variables are shown in Table 3. We assume the initial vector population is the double of the human population, according to the work of Menach et al. [24]. The algorithm of the simulation can be found in Additional file 2.

According to these values, the initial values of *p* and *q* would be 0.375 and 0.625, respectively.

A total of three main simulations were made, each one with different insecticide resistance situations. In the first one, we considered that both alleles (*a* and *A*) conferred a mortality rate of 0.25day^−1^. In this case, we intended to simulate the Hardy-Weinberg equilibrium conditions, in which all the individuals of a population have the same chance of surviving and reproducing, regardless of its genotype. This situation would also represent the absence of an insecticide-resistant strain, *i.e.*, a population totally susceptible to insecticides. In the other two simulations, we aimed to simulate the presence of an insecticide-resistant strain and the natural selection, which is characterized by differential reproduction and survival of organism. For this reason, in the second simulation, the recessive allele attributed a smaller mortality value, while the dominant allelomorph conferred a higher rate. Finally, the last simulation was similar, but then the dominant allele conferred a greater fitness. The values of the mortality rates can be seen in Table 4.

We also did four other minor simulations. One of them was similar to simulation 1, but the time ranged from *t*=0 to *t*=500. The other three simulations were analogous to the three main simulations, except by the fact that the initial values of variables were ten times smaller than the values shown in Table 3, and *Δ* *t*=0.01. These simulations with smaller populations are referred as 1s, 2s and 3s. 1s, 2s and 3s have, respectively, no insecticide-resistant gene, recessive insecticide resistance gene and dominant insecticide resistance gene.

In the outputs of all simulations, genotypes *AA* and *Aa* were represented together as *A*_, since they express the same phenotype.

## Results

### Simulation 1

**Fig. 3:**
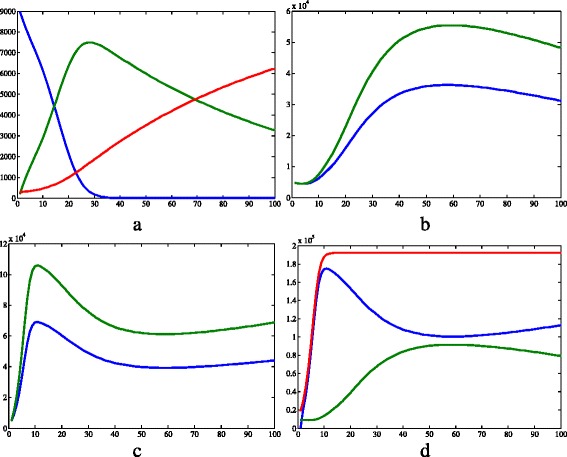
Results of simulation 1. **a** Susceptible (*blue*), infected (*green*) and recovered humans (*red*). **b** Infected *aa* (*blue*) and *A*_ (*green*) vectors. **c** Susceptible *aa* (*blue*) and *A*_ (*green*) vectors. **d** Susceptible (*blue*), infected (*green*) and total (*red*) populations of vectors disregarding the genetic separation. *c*=0.457·10^−4^, *μ*_*h*_=0.457·10^−4^, *β*_*h*_=0.4, *γ*=0.121, *θ*=6.353, *β*_*v*_=0.4, *K*=2·10^5^, *μ*_*aa*_=*μ*_*Aa*_=*μ*_*AA*_=0.25, *S*_*h*_=9·10^3^, *I*_*h*_=2·10^2^, *R*_*h*_=3·10^2^, *N*_*h*_=9.5·10^3^, *S*_*Aa*_=2.375·10^3^, *I*_*Aa*_=2.375·10^3^, *N*_*Aa*_=4.75·10^3^, *S*_*aa*_=4.75·10^3^, *I*_*aa*_=4.75·10^3^, *N*_*aa*_=9.5·10^3^, *S*_*AA*_=2.375·10^3^, *I*_*AA*_=2.375·10^3^, *N*_*AA*_=4.75·10^3^, *I*_*v*_=3·104, *N*_*v*_=1.9·10^4^. *X axis* represents time (days) and *Y axis* represents number of individuals. Image produced with MatLab^®;^

**Fig. 4:**
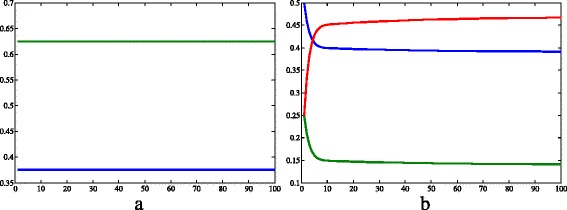
Results of simulation 1 (continued). **a** Allele frequencies of *a* (*green*) and *A* (*blue*). **b** Genotype frequencies of *aa* (*blue*), *Aa* (*red*) and *AA* (*green*). *c*=0.457·10^−4^, *μ*_*h*_=0.457·10^−4^, *β*_*h*_=0.4, *γ*=0.121, *θ*=6.353, *β*_*v*_=0.4, *K*=2·10^5^, *μ*_*aa*_=*μ*_*Aa*_=*μ*_*AA*_=0.25, *S*_*h*_=9·10^3^, *I*_*h*_=2·10^2^, *R*_*h*_=3·10^2^, *N*_*h*_=9.5·10^3^, *S*_*Aa*_=2.375·10^3^, *I*_*Aa*_=2.375·10^3^, *N*_*Aa*_=4.75·10^3^, *S*_*aa*_=4.75·10^3^, *I*_*aa*_=4.75·10^3^, *N*_*aa*_=9.5·10^3^, *S*_*AA*_=2.375·10^3^, *I*_*AA*_=2.375·10^3^, *N*_*AA*_=4.75·10^3^, *I*_*v*_=3·10^4^, *N*_*v*_=1.9·10^4^. *X axis* represents time (days) and *Y axis* represents freqquency.Image produced with MatLab^®;^

Figures 3 and 4 show the graphs obtained from simulation 1, in which we aim to represent the Hardy-Weinberg equilibrium conditions, and a population without resistant strains. In Fig. 3 a, the human populations are represented. The number of susceptible humans decreases until it reaches the value zero, while the population of recovered humans increases almost linearly. On the other hand, the population of infected humans raised to a peak at time 28 and then diminished. In relation to the vectors (Fig. 3 b, c and d), their populations exhibited both periods of growth and decline. The proportion of *A*_ vectors were always greater than the proportion of recessive homozygotes, except in the initial time, when their values were the same.

The frequencies of the dominant (*p*=0.375) and the recessive allele (*q*=0.625) remained constant during the whole time considered, as expected for the absence of evolution factors. According to Fig. 4b, the genotype frequencies varied very fast at the beginning of the simulation, and then they started to increase or decrease very slowly. The Hardy-Weinberg equilibrium would be achieved when:

$$\begin{array}{*{20}l} f(aa)=q^{2}=0.390625 \\ f(Aa)=2\cdot p\cdot q=0.468750 \\ f(AA)=p^{2}=0.140625 \end{array} $$

According to another simulation with the same conditions, but in a longer time frame, the Hardy-Weinberg equilibrium is achieved at *t*=488. Note that *f*(*a* *a*)+*f*(*A* *a*)+*f*(*A* *A*)=1.

### Simulation 2

**Fig. 5:**
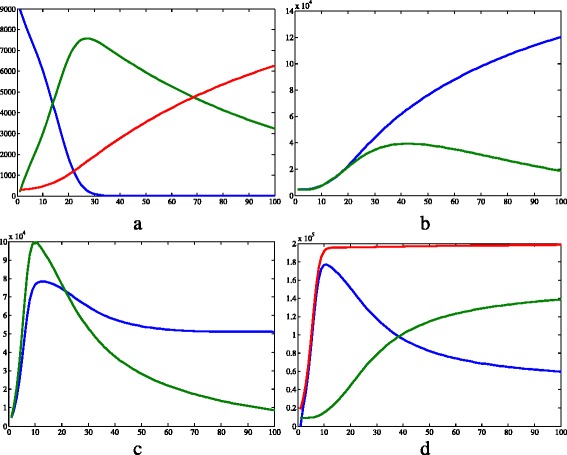
Results of simulation 2. **a** Susceptible (*blue*), infected (*green*) and recovered humans (*red*). **b** Infected *aa* (*blue*) and *A*_ (*green*) vectors. **c** Susceptible *aa* (*blue*) and *A*_ (*green*) vectors. **d** Susceptible (*blue*), infected (*green*) and total (*red*) populations of vectors disregarding the genetic separation. *c*=0.457·10^−4^, *μ*_*h*_=0.457·10^−4^, *β*_*h*_=0.4, *γ*=0.121, *θ*=6.353, *β*_*v*_=0.4, *K*=2·10^5^, *μ*_*aa*_=0.01, *μ*_*Aa*_=*μ*_*AA*_=0.25, *S*_*h*_=9·10^3^, *I*_*h*_=2·10^2^, *R*_*h*_=3·10^2^, *N*_*h*_=9.5·10^3^, *S*_*Aa*_=2.375·10^3^, *I*_*Aa*_=2.375·10^3^, *N*_*Aa*_=4.75·10^3^, *S*_*aa*_=4.75·10^3^, *I*_*aa*_=4.75·10^3^, *N*_*aa*_=9.5·10^3^, *S*_*AA*_=2.375·10^3^, *I*_*AA*_=2.375·10^3^, *N*_*AA*_=4.75·10^3^, *I*_*v*_=3·10^4^, *N*_*v*_=1.9·10^4^. *X axis* represents time (days) and *Y axis* represents number of individuals.Image produced with MatLab^®;^

**Fig. 6:**
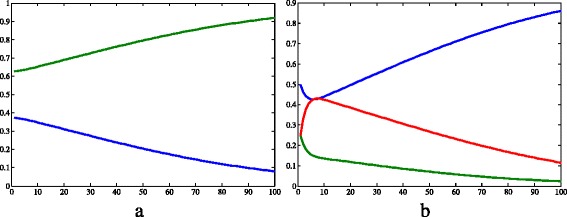
Results of simulation 2 (continued). **a** Allele frequencies of *a* (*green*) and *A* (*blue*). **b** Genotype frequencies of *aa* (*blue*), *Aa* (*red*) and *AA* (*green*). *c*=0.457·10^−4^, *μ*_*h*_=0.457·10^−4^, *β*_*h*_=0.4, *γ*=0.121, *θ*=6.353, *β*_*v*_=0.4, *K*=2·10^5^, *μ*_*aa*_=0.01, *μ*_*Aa*_=*μ*_*AA*_=0.25, *S*_*h*_=9·10^3^, *I*_*h*_=2·10^2^, *R*_*h*_=3·10^2^, *N*_*h*_=9.5·10^3^, *S*_*Aa*_=2.375·10^3^, *I*_*Aa*_=2.375·10^3^, *N*_*Aa*_=4.75·10^3^, *S*_*aa*_=4.75·10^3^, *I*_*aa*_=4.75·10^3^, *N*_*aa*_=9.5·10^3^, *S*_*AA*_=2.375·10^3^, *I*_*AA*_=2.375·10^3^, *N*_*AA*_=4.75·10^3^, *I*_*v*_=3·10^4^, *N*_*v*_=1.9·10^4^. *X axis* represents time (days) and *Y axis* represents freqquency. Image produced with MatLab^®;^

The results of simulation 2, in which the recessive allele conferred a greater fitness, are shown in Figs. 5 and 6. The recessive homozygotes represent the resistant strain, which is positively selected.

Regarding the humans (Fig. 5a), the susceptible population decreased during the whole time considered, until the zero value. The infected population increased until time 27 of the simulation and then reduced up to the end of simulation. Finally, the recovered population had an approximately linear growth. This human dynamics is approximately similar to simulation 1, although the values vary lightly. On the other hand, the vector population showed different dynamics (Fig. 5b and c). The dynamics of the susceptible vectors (Fig. 5c) can be split into two moments. In the first one, the populations increased quickly and the proportion of *A*_ individuals was greater than that of *aa* individuals. At a second moment, the number of *A*_ and *aa* susceptible vectors decreased and the proportion of pure recessives overcame the proportion of the other genotypes. The number of pure recessive infected vectors all increased over the time, while the quantity of infected individuals that carry the dominant allele had periods of growth and decline.

The recessive allele tended to fixation while the frequency of the dominant one decreased over time (Fig. 6a). According to another simulation with the same conditions, but a bigger time, fixation occurs when *t*=722. Despite the beginning of the simulation, the frequency of the genotype *aa* increased, while the frequency of the others decreased.

### Simulation 3

**Fig. 7:**
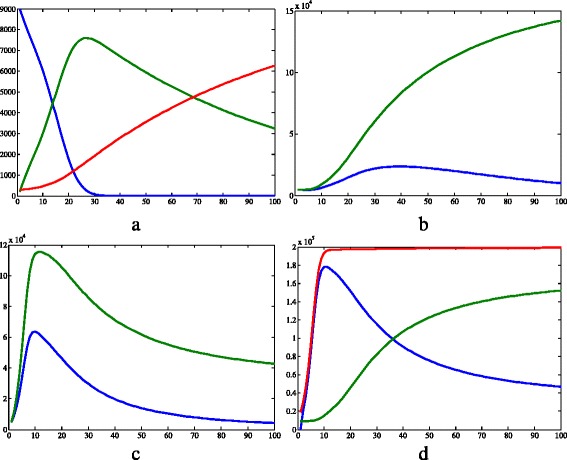
Results of simulation 3. **a** Susceptible (*blue*), infected (*green*) and recovered humans (*red*). **b** Infected *aa* (*blue*) and *A*_ (*green*) vectors. **c** Susceptible *aa* (*blue*) and *A*_ (*green*) vectors. **d** Susceptible (*blue*), infected (*green*) and total (*red*) populations of vectors disregarding the genetic separation. *c*=0.457·10^−4^, *μ*_*h*_=0.457·10^−4^, *β*_*h*_=0.4, *γ*=0.121, *γ*=6.353, *β*_*v*_=0.4, *K*=2·10^5^, *μ*_*aa*_=0.25, *μ*_*Aa*_=*μ*_*AA*_=0.01, *S*_*h*_=9·10^3^, *I*_*h*_=2·10^2^, *R*_*h*_=3·10^2^, *N*_*h*_=9.5·10^3^, *S*_*Aa*_=2.375·10^3^, *I*_*Aa*_=2.375·10^3^, *N*_*Aa*_=4.75·10^3^, *S*_*aa*_=4.75·10^3^, *I*_*aa*_=4.75·10^3^, *N*_*aa*_=9.5·10^3^, *S*_*AA*_=2.375·10^3^, *I*_*AA*_=2.375·10^3^, *N*_*AA*_=4.75·10^3^, *I*_*v*_=3·10^4^, *N*_*v*_=1.9·10^4^. *X axis* represents time (days) and *Y axis* represents number of individuals. Image produced with MatLab^®;^

**Fig. 8:**
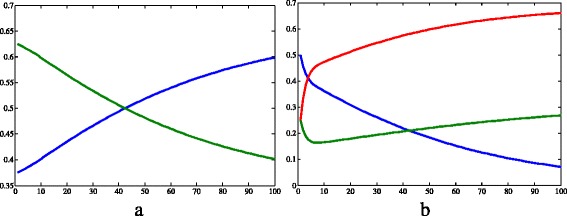
Results of simulation 3 (continued). **a** Allele frequencies of *a* (*green*) and *A* (*blue*). **b** Genotype frequencies of *aa* (*blue*), *Aa* (*red*) and *AA* (*green*). *c*=0.457·10^−4^, *μ*_*h*_=0.457·10^−4^, *β*_*h*_=0.4, *γ*=0.121, *θ*=6.353, *β*_*v*_=0.4, *K*=2·10^5^, *μ*_*aa*_=0.25, *μ*_*Aa*_=*μ*_*AA*_=0.01, *S*_*h*_=9·10^3^, *I*_*h*_=2·10^2^, *R*_*h*_=3·10^2^, *N*_*h*_=9.5·10^3^, *S*_*Aa*_=2.375·10^3^, *I*_*Aa*_=2.375·10^3^, *N*_*Aa*_=4.75·10^3^, *S*_*aa*_=4.75·10^3^, *I*_*aa*_=4.75·10^3^, *N*_*aa*_=9.5·10^3^, *S*_*AA*_=2.375·10^3^, *I*_*AA*_=2.375·10^3^, *N*_*AA*_=4.75·10^3^, *I*_*v*_=3·10^4^, *N*_*v*_=1.9·10^4^. *X axis* represents time (days) and *Y axis* represents freqquency. Image produced with MatLab^®;^

The graphs in Figs. 7 and 8 show the results of the third simulation, in which the dominant allele conferred insecticide resistance (*i.e.*, a smaller mortality value), therefore the resistant strain consists of *AA* and *Aa* individuals. Regarding the human population (Fig. 7a), the number of susceptible individuals decrease until reaching the value zero, while the quantity of recovered grew during the whole time considered. On the other hand, the infected population increased to a maximal at day 27 and then started to go down. Concerning the vectors, the dominant homozygotes and heterozygotes were more numerous than the recessive homozygotes throughout the whole simulation (Fig. 7b and c), except for the initial time. As for the infected compartment (Fig. 7b), the *A*_ compartment increased, while the group of pure recessives barely varied over the whole time considered. The susceptible vectors (Fig. 7) had an initial period of growth followed by a period of decrease, and the *A*_ susceptible vectors were more numerous than the *aa* ones. The population of susceptible vectors grew until day 11, when it started to decrease (Fig. 7d), and then was eventually overcome by the infected population.

Finally, regarding the allelic frequencies, the dominant allele tended to fixation, while the recessive tended to zero (Fig. 8a). In an attempt to find when the allele *A* would fix in the population, we did a similar computational simulation over time varying from 0 to 100000, but even in these conditions the fixation was not yet reached. Figure 8b shows that the frequencies of heterozygotes and pure dominants, which had a bigger fitness, increased; while the frequency of pure recessive, that had a smaller fitness, decreased.

### Simulations with bigger time interval and smaller population

**Fig. 9:**
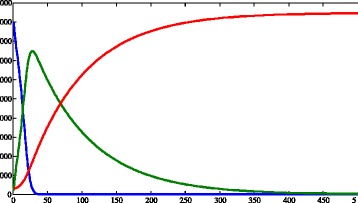
Simulation with the same conditions to first simulation, with time varying from 0 to 500. Legend: Susceptible (*blue*), infected (*green*) and recovered humans (*red*). *c*=0.457·10^−4^, *μ*_*h*_=0.457·10^−4^, *β*_*h*_=0.4, *γ*=0.121, *θ*=6.353, *β*_*v*_=0.4, *K*=2·10^5^, *μ*_*aa*_=*μ*_*Aa*_=*μ*_*AA*_=0.25, *S*_*h*_=9·10^3^, *I*_*h*_=2·10^2^, *R*_*h*_=3·10^2^, *N*_*h*_=9.5·10^3^, *S*_*Aa*_=2.375·10^3^, *I*_*Aa*_=2.375·10^3^, *N*_*Aa*_=4.75·10^3^, *S*_*aa*_=4.75·10^3^, *I*_*aa*_=4.75·10^3^, *N*_*aa*_=9.5·10^3^, *S*_*AA*_=2.375·10^3^, *I*_*AA*_=2.375·10^3^, *N*_*AA*_=4.75·10^3^, *I*_*v*_=3·10^4^, *N*_*v*_=1.9·10^4^. *X axis* represents time (days) and *Y axis* represents number of individuals. Image produced with MatLab^®;^

Figure 9 shows the results for the simulation in interval 0<*t*<500. Unlike the preceding simulations, it enables us to see that the infected human population almost reaches the value zero, when all the human compartments practically arrive at a stable value (*i.e.*, reaching the equilibrium).

**Fig. 10:**
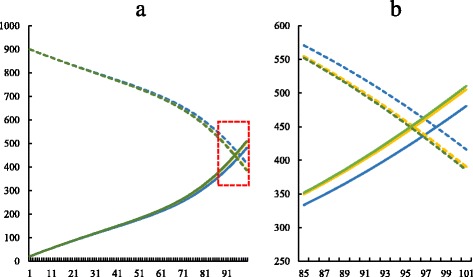
Smaller initial values. Simulations with the same conditions as the 1st, 2nd and 3rd simulations, but with initial values of variables ten times smaller than shown in Table 2. **a** complete charts of the simulation. The dashed square marks the region represented by the figure on the right. **b** zoom on the left figure. *Dashed lines* = susceptible humans; *continuous lines* = infected humans; *blue lines* = conditions similar to the first simulation; *yellow lines* = conditions similar to the second simulation; *green lines* = conditions similar to the third simulation. Graphics produced on Microsoft Excel^®;^ with data obtained on MatLab^®;^ simulations. *X axis* represents time (days) and *Y axis* represents number of individuals

On the other hand, Fig. 10 shows the dynamics of humans populations of 1s, 2s, and 3s. The results show a slight difference between them. In the presence of an allele that confers resistance, the number of infected people is bigger and the number of susceptible, smaller. Conversely, when there are no resistant strains, the number of infected humans is smaller and the susceptible compartment is bigger.

## Discussion

In this study, we propose a SIR model that considers the population genetics of vector insecticide resistance. We did three computational simulations: in one of them, all vectors of the population had the same insectide-induced mortality, so that there was no selection and no resistant strain. In the second, the recessive homozygotes vectors were resistant to an insecticide, and therefore they had a smaller mortality rate and a bigger fitness. In the third, those who carried the dominant allele had insecticide resistance. Therefore, they had more probability of passing their genes to the next generation. Our model is based on the biology of several vector-borne diseases such as dengue fever, yellow fever and chikungunya. All of them are caused by viruses of the genus *Flavivirus* and are transmitted by *Aedes* spp., especially *Aedes aegypti* [25–27].

These tropical neglected diseases are extremely important nowadays: in Brazil, until the 21st epidemiological week of 2016, almost 1.3 million people came down with dengue and 122762 probable cases of chikungunya were registered [28]; in one year, 60000 people died because of yellow fever in Sub-Saharian Africa [29]. However, the present model must be interpreted with caution because it disregards important factors, such as seasonal variation of temperature [19], different life stages of the vector [19, 20], spatial distribution of populations [21] and the existence of various strains of a pathogen [30]. Therefore, it is important to bear in mind that the computational simulations may fail in accurately representing all aspects of a real epidemics. Notwithstanding these restrictions, our model is important for understanding the influence of insecticide resistance genetics on dynamics of an epidemics, without influence of other factors. In addition, it may serve as a basis for the development of more complex and realistic approaches.

In our model, we considered a pair of allelomorphs with complete dominance interaction and Mendelian inheritance, which affected the mortality rate of the vector. Using this approach, we intended to simulate a genetic-induced insecticide resistance. Several similar real cases are described in the literature. According to Saavedra-Rodriguez et al. [31], an isoleucine replacement in codon 1016 of voltage-gated sodium channel transmembrane protein gene of *Aedes aegypti* is linked to knockdown resistance to pyrethroids, which is a very common type of insecticide. The allele that contains this mutation is recessive and increased in frequency when under insecticide selection [31]. Another interesting example is found in the study by Bonin et al. [32], who researched some alleles in *Aedes aegypti* related to resistance to the bioinsecticide *Bacillus thuringiensis israelensis*. These allelomorphs have dominant characteristics. However, the genes considered in that study [32] are poligenes, *i.e.*, they determine a quantitative character, which is different from our mathematical model, which considers that insecticide resistance is qualitative. There are many different mutations of this kind in several mosquito populations around the world [33].

Analyzing Figs. 3d, 5d and 7d, it is clear that the total vector population is smaller in the first simulation than in the others. This can be easily explained by the fact that more individuals are removed there, since the mean mortality rate is bigger because all vectors have a mortality value of 0.25day^−1^. In contrast, in the other two simulations, there is a portion of the population with *μ* *T*=0.01day^−1^, and therefore the mean mortality is smaller and the total number of vectors is bigger.

**Fig. 11:**
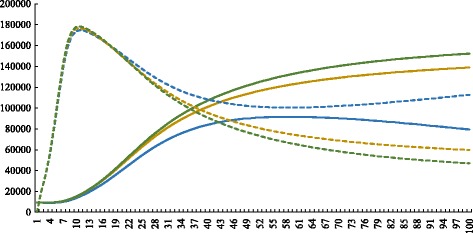
Total infected (*continuous lines*) and total susceptible (*dashed lines*) vectors in simulations 1, 2 and 3. *Blue* = First simulation; *yellow* = second simulation; *green* = third simulation. Graphics produced on Microsoft Excel^®;^, but with data obtained on MatLab^®;^ simulations. *X axis* represents time (days) and *Y axis* represents number of individuals

Another interesting observation to make about Figs. 3d, 5d and 7d is that when there is an insecticide-resistant strain, the proportion of vectors infected with the pathogen is bigger. In addition, this proportion is even bigger when the resistance is caused by a dominant allele instead of a recessive one. This can be easily seen in Fig. 11. One possible explanation for this phenomenon is that when there is a resistant strain, the mean mortality rate is smaller, as stated above in this section. Therefore, less susceptible vectors die, and this compartment become more numerous.

According to the expression $\frac {\beta _{v}I_{h}}{N_{h}}\cdot S_{v}$ of system 11, the bigger the number of susceptible vectors, the more they become infected and the bigger the compartment of infected vectors. The larger proportion of infected vectors in the presence of resistance was also observed in laboratory experiments in vivo. For instance, Labout et al. [34] studied three strains of *Anopheles gambiae*, the malaria vector: all of them had the same genetic background, despite the fact that one group was susceptible to insecticides, another had a mutation that conferred resistance to organophosphates and carbamates; and the third had a knockdown mutation that determined resistance to pyrethroids and DDT. The mosquitoes were fed on blood with *Plasmodium falciparum*; the protozoan that causes malaria, and then the prevalence of *P. falciparum* infection in the vectors strains was measured. They found that the prevalence was higher in insecticide resistant strains than in the susceptible one. Alout et al. [34] explain this phenomenon through a molecular mechanism, while our present paper justify it through population dynamics. Probably, during an epidemic, both mechanisms influence the proportion of infected vectors.

When the outputs of the three simulations are compared, a slight difference between the human population dynamics can be observed. This difference is more visible when there are less individuals (Fig. 10). It can be noted that the number of infected people is greater in presence of resistant vectors, and smaller in their absence. The inverse is valid for susceptible humans. This might happen because of the larger proportion of infected vectors in simulations 2s and 3s and smaller in simulation 1s, in conformity to the statements of the previous paragraph. The more vectors are infected, the faster humans become infected. The bigger is the compartment of infected people, the smaller is the compartment of susceptible people.

Although we diminished the populations in the simulation in Fig. 10 and it had interesting results, in real situations the natural selection becomes less relevant in small populations, in which the genetic drift is the most actable evolution factor [15]. For this reason, we considered greater variable values in the other simulations, even though the effect in humans was more subtle. One can argue that the differences between the simulations of Fig. 10 are a bias due to the small size of populations. As a matter of fact, in vivo and in vitro approaches that involve small samples usually face this issue. However, our in silico study used only non-stochastic mathematical tools, so that the results are always exact or depends only on the variables and parameters considered. In order to confirm this, the computational simulations were performed several times, and all of them presented the same results. Therefore, the differences in human populations did not occur because of the small population size, but because of the genetics of the vector insecticide resistance.

Taken together, the results of simulations 1, 2, 3, 1s, 2s and 3s suggest that the genetics of insecticide resistance have stronger effect on the vector population than in the human population. This result may be explained by the fact that the insecticide resistance gene have direct effect on the vectors populations, but only indirectly influences the human populations. As a matter of fact, the insecticide resistance have direct effect on vector mortality rate, which influences the size of susceptible and infected vector populations. In turn, changes in vector infected population causes changes in human susceptible and infected populations, that are also restrained by *β*_*h*_ and *N*_*h*_. Indeed, in simulations 2, 3, 2s and 3s, the differences in genotype frequencies among the simulations can be noticed in the first days of simulation (Figs. 6b, 8b), the differences in size of vector infected populations can be seen from day 20 on (Fig. 11), while the differences in size of human infected populations only can be noticed from day 80 on (Fig. 10b). Overall, this information show that the genetics of the vectors affects the epidemiological dynamics of a vector-borne disease as to several important aspects. Therefore, the present model could be an important start point for investigating population dynamics in epidemics.

Another relevant observation is that, in all simulations of our model, the populations of susceptible humans always decreased and reached zero, and never went up. This happens because of the combination of two factors: (1) the value of the human birth rate is equal to the value of the human mortality rate, and (2) the immunity of the recovered was lifelong, so they would never become susceptible again. This situation could be reversed by considering different values of *c* and *μ*_*h*_, but it was decided to use the same values for both parameters in order to maintain constant the size of total human population during all the time of simulation. It was decided to consider lifelong immunity because this characteristic is observed for important vector-borne diseases such as chikungunya [35] and for each dengue serotype [36].

It is interesting to note that the curve obtained for infected humans in all three computational simulations has a very similar shape to many line graphs made with real epidemiological data. For example, we can cite the Brazilian dengue epidemics in the years of 2013, 2014, and 2015, whose line graphs for the number of infected people over time can be found in the epidemiological bulletins of the Brazilian Secretary of Health Vigilance [28]. In all of them, during the rainy season, the number of infected people increases until it reaches a peak, and then it starts to decrease, similar to the outputs obtained here. The same trend is observed in the number of yellow fever suspect cases in Angola, from December, 2015 to March, 2016 [2].

Regarding the allele frequencies, in all simulations, the initial *p* and *q* were 0.375 and 0.625, respectively. However, these variables changed very differentially according to the simulation. As stated in the results of simulation 1, in the first simulation, the frequencies did not change because of the lack of evolutionary factors. Comparing simulations 2 and 3, we can infer that the resistance allele fixes faster when this allele is recessive than when it is dominant: the time it takes to reach the frequency 100% is longer when *A* confers resistance; and the variation of frequency between 0<*t*<100 is smaller in the third simulation (Table 5). According to Freeman and Herron [15], this happens because, when a dominant allele is positively selected, the recessive allele that confer a smaller fitness is maintained through the heterozygotes, that “hide” the recessive alleles, since they carry them but express the phenotype of bigger fitness. When the recessive allelomorph is positively selected, the situation is different: the heterozygotes have a smaller fitness despite the fact they carry the two forms of the gene, and therefore they tend to be eliminated from the population.

**Table 5:** Initial and final allele frequencies in the simulation

Simulation	*p*			*q*		
	Initial	Final	Initial-final	Initial	Final	Initial-final
1^*st*^	0.375	0.375	0	0.625	0.625	0
2^*nd*^	0.375	0.081	0.294	0.625	0.919	0.294
3^*rd*^	0.375	0.599	0.224	0.625	0.401	0.224

The frequencies of the genotypes in simulations 2 and 3, at the beginning of the time considered, have similar dynamics to the first simulation. After this initial period, they start to vary according to the selective pressure.

## Conclusion

In conclusion, the model presented in this work exhibited results in conformation with the literature, and the numerical results show that the genetics of the vectors do influence human population dynamics. The computational simulations have shown that the presence of an insecticide resistance gene is related to a bigger number of infected humans and vectors. The numerical investigation has also shown that this aspect depends on the inheritance pattern of the gene, since a dominant insecticide resistance gene implies in an even greater number of infected people and vectors.

This study does not consider factors such as different life stages of vectors and the possibility of diverse serotypes of a pathogen. Notwithstanding these restrictions, our model could be a basis for the development of more sophisticated epidemiological models that considers insecticide resistance genetics. These, in turn, could be used for monitoring diseases such as yellow fever and predicting trends of epidemics. This way, the present investigation open new perspectives for further studies. For example, our model could be a starting point to calculate the optimum control in which insecticides are used in a manner that minimizes the evolution of resistance in vector population.

## Additional files

Additional file 1 The Discretized form of the model obtained through the Euler method that was used for the numerical simulations. (PDF 95 kb)

Additional file 2 Algorithim used for the numerical simulations of the mathematical models that indicates the steps necessary to obtain the results. (PDF 69 kb)

## Abbreviations

*K*: Carrying capacity
*A*: Dominant allele
*μ*_*AA*_: Dominant homozygote vector mortality rate
*S*_*AA*_: Dominant homozygote vector susceptible population
*I*_*AA*_: Dominant homozygote vector infected population
*N*_*AA*_: Dominant homozygote vector total population
*p*: Frequency of the dominant allele in the population
*q*: Frequency of the recessive allele in the population
*Aa*: Heterozygote
*c*: Human birth rate
*μ*_*h*_: human mortality rate
*γ*: Human recovery rate
*μ*_*Aa*_: Heterozygote vector mortality rate
*S*_*h*_: Human susceptible population
*I*_*h*_: Human infected population
*R*_*h*_: Human recovered population
*N*_*h*_: Human total population
*S*_*Aa*_: Heterozygote vector susceptible population
*I*_*Aa*_: Heterozygote vector infected population
*N*_*Aa*_: Heterozygote vector total population
*I*_*v*_: Infected vector population
*aa*: Pure recessive
*AA*: Pure dominant
*A*_: Pure dominants and heterozygotes
*β*_*h*_: probability of vector-human transmission
*β*_*v*_: Probability of human-vector transmission
*a*: Recessive allele
*μ*_*aa*_: Recessive homozygote vector mortality rate
*S*_*aa*_: Recessive homozygote vector susceptible population
*I*_*aa*_: Recessive homozygote vector infected population
*N*_*aa*_: Recessive homozygote vector total population
*SIR*: Susceptible-Infected-Recovered
*N*_*v*_: Vector total population
*θ*: Vector oviposition rate
