# Supplementary material for: Mathematical modelling of vector-borne diseases and insecticide resistance evolution
Source: J Venom Anim Toxins Incl Trop Dis. 2017 Jul 6;23:34. doi: 10.1186/s40409-017-0123-x (PMC5501426; doi:10.1186/s40409-017-0123-x)
Supplement: Supplementary file 1 — The Discretized form of the model obtained through the Euler method that was used for the numerical simulations. (PDF 95 kb) [file 40409_2017_123_MOESM1_ESM.pdf]

Discretized form of the model

Applying the Euler method in ODE system 11, we obtain the discrete form as described below. Using the discrete form (1) in the numerical simulations, we can finally obtain the numerical results to the vector-borne diseases, in which the Population Genetics of vector insecticide resistance is considered.

For $t = T_0$ **to** $T_f - 1$**:**

$$
\begin{cases}
{S_h}^{t+1} &= {S_h}^{t} + \Delta t\Bigg(cN_h^t - \left(\frac{\beta_h I_v}{N_h^t} + \mu_h\right) S_h^t\Bigg) \\
{I_h}^{t+1} &= {I_h}^{t} + \Delta t\Bigg(\frac{\beta_h I_v}{N_h^t} S_h^t - (\gamma + \mu_h)\, I_h^t\Bigg) \\
{R_h}^{t+1} &= {R_h}^{t} + \Delta t\Bigg(\gamma I_h^t - \mu_h R_h^t\Bigg) \\
{N_h}^{t+1} &= {S_h}^{1+t} + {I_h}^{t+1} + {R_h}^{t+1} \\
{S_{aa}}^{t+1} &= {S_{aa}}^{t} + \Delta t\Bigg(\left(\frac{(2N_{aa}^t + N_{Aa}^t)}{2N_v^t}\right)^2 \theta N_v^t \left(1 - \frac{N_v^t}{K}\right) - \left(\frac{\beta_v I_h^t}{N_h^t} + \mu_{aa}\right) S_{aa}^t\Bigg) \\
I_{aa}^{t+1} &= I_{aa}^t + \Delta t\Bigg(\frac{\beta_v I_h^t}{N_h^t} S_{aa}^t - \mu_{aa} I_{aa}^t\Bigg) \\
N_{aa}^{t+1} &= S_{aa}^{t+1} + I_{aa}^{t+1} \\
S_{AA}^{t+1} &= S_{AA}^t + \Delta t\Bigg(\left(\frac{(2N_{AA}^t + N_{Aa}^t)}{2N_v^t}\right)^2 \theta N^t \Bigg(1 - \frac{N^t}{K}\Bigg) - \Bigg(\frac{\beta_v I_h^t}{N_h^t} + \mu_{AA}\Bigg) S_{AA}^t\Bigg) \\
I_{AA}^{t+1} &= I_{AA}^t + \Delta t\Bigg(\frac{\beta_v I_h^t}{N_h^t} S_{AA}^t - \mu_{AA} I_{AA}^t\Bigg) \\
N_{AA}^{t+1} &= S_{AA}^{t+1} + I_{AA}^{t+1} \\
S_{Aa}^{t+1} &= S_{Aa}^t + \Delta t\Bigg(\Bigg(2\frac{(2N_{aa}^t + N_{Aa}^t)}{2N_v^t}\frac{(2N_{AA}^t + N_{Aa}^t)}{2N_v^t}\Bigg)\theta N_v^t \left(1 - \frac{N_v^t}{K}\right) - \left(\frac{\beta_v I_h^t}{N_h^t} + \mu_{Aa}\right) S_{Aa}^t\Bigg) \\
I_{Aa}^{t+1} &= I_{Aa}^t + \Delta t\Bigg(\frac{\beta_v I_h}{N_h} S_{AA} - \mu_{AA} I_{AA}\Bigg) \\
N_{Aa}^{t+1} &= S_{Aa}^{t+1} + I_{Aa}^{t+1} \\
N^{t+1} &= N_{aa}^{t+1} + N_{AA}^{t+1} + N_{Aa}^{t+1} \\
S^{t+1} &= S_{aa}^{t+1} + S_{Aa}^{t+1} + S_{AA}^{t+1} \\
I^{t+1} &= I_{aa}^{t+1} + I_{AA}^{t+1} + I_{Aa}^{t+1} \\
S_{A\_}^{t+1} &= S_{AA}^{t+1} + S_{Aa}^{t+1} \\
I_{A\_}^{t+1} &= I_{AA}^{t+1} + I_{Aa}^{t+1} \\
F_{aa}^{t+1} &= \frac{N_{aa}^{t+1}}{N^{t+1}} \\
F_{Aa}^{t+1} &= \frac{N_{Aa}^{t+1}}{N^{t+1}} \\
F_{AA}^{t+1} &= \frac{N_{AA}^{t+1}}{N^{t+1}}
\end{cases}
\tag{1}
$$
