# Supplementary material for: Mathematical modelling of vector-borne diseases and insecticide resistance evolution
Source: J Venom Anim Toxins Incl Trop Dis. 2017 Jul 6;23:34. doi: 10.1186/s40409-017-0123-x (PMC5501426; doi:10.1186/s40409-017-0123-x)
Supplement: Supplementary file 2 — Algorithim used for the numerical simulations of the mathematical models that indicates the steps necessary to obtain the results. (PDF 69 kb) [file 40409_2017_123_MOESM2_ESM.pdf]

Algorithm

The updated populations SIR for humans and SI for mosquitos at the time $t+1$ is calculated with the following sequencies of steps:

Step 1: Input the biogical parameters;
Step 2: Set the initial conditions for all state variables *SIRSI*;
Step 3: Define the time-step dt to the numerical method;
Step 4: Set the initial and final times: T_0 and T_f;
Step 5: For $t = T_0$ to T_f:

- Compute the populations $SIRSI$ from a discretized form of the ODE system 11 by Euler method, using the dt defined in **step 5**;
- Update all frequencies $f(aa)$, $f(Aa)$, $f(AA)$, p and q;

Step 6: Check the *Hardy-Weinberg* equilibrium, in which $f(aa) = q^2$, $f(Aa) = 2pq$ and $f(AA) = p^2$.
Step 8: Check the differences of the dynamics of the three genotypes.
